# The Impact of Study-at-Home During the COVID-19 Pandemic on Myopia Progression in Chinese Children

**DOI:** 10.3389/fpubh.2021.720514

**Published:** 2022-01-06

**Authors:** Dandan Ma, Shifei Wei, Shi-Ming Li, Xiaohui Yang, Kai Cao, Jianping Hu, Xiaoxia Peng, Ruohua Yan, Jing Fu, Andrzej Grzybowski, Zi-Bing Jin, Ningli Wang

**Affiliations:** ^1^Beijing Institute of Ophthalmology, Beijing Tongren Eye Center, Beijing Tongren Hospital, Capital Medical University, Beijing, China; ^2^Center for Clinical Epidemiology and Evidence-based Medicine, Beijing Children's Hospital, Capital Medical University, Beijing, China; ^3^University of Warmia and Mazury, Olsztyn, Poland; ^4^Institute for Research in Ophthalmology, Poznan, Poland

**Keywords:** study-at-home, COVID-19, children, myopia progression, cycloplegic spherical equivalent

## Abstract

**Background:** To assess the impact of study-at-home during the COVID-19 pandemic on myopia development in Chinese schoolchildren.

**Methods:** This historical cohort involved two groups with a total of 154 children. The exposed group was formed from 77 children aged 8 to 10 years who studied at home in the 7-month period during the COVID-19 pandemic (follow-up period: January – August 2020) and did not study at home in the 7-month period before the COVID-19 outbreak (baseline period: July 2019 – January 2020). Seventy-seven children who did not undergo study-at-home (baseline period: 7 months in 2015, follow-up period: 7 months in 2016) were included in the control group. Cycloplegic refraction, axial length and uncorrected visual acuity were measured 3 times. The questionnaire mainly focused on collecting visual habits.

**Results:** Myopia progression was similar between the two groups in the baseline period. However, in the follow-up period the exposed group had a greater change in refraction toward myopia (−0.83 ± 0.56 D) than the control group (−0.28 ± 0.54 D; *p* < 0.001). In addition, the exposed group exhibited a significantly greater change in refraction toward myopia in the follow-up period (−0.83 ± 0.56 D) than in the baseline period (−0.33 ± 0.46 D; *p* < 0.001). Difference-in-difference analysis indicated that study-at-home accelerated the change in refraction toward myopia (*t* = −0.567; *p* < 0.001).

**Conclusions:** During the COVID-19 pandemic study-at-home accelerated the change of refraction toward myopia in children.

## Background

The novel coronavirus disease 2019 (COVID-19) has had a global impact (1) and has affected many aspects of people's lives. During the COVID-19 pandemic, governments around the world provided distance education for children to study at home. According to the United Nations, the COVID-19 pandemic has created the largest disruption of education systems in history, affecting nearly 1.6 billion learners in more than 190 countries and all continents (2). From January 2020 to August 2020, the Ministry of Education of the People's Republic of China estimated that more than 220 million children and adolescents stayed home and engaged in online education in China (3). Previous studies have reported that closures of schools and studying at home may have elevated psychosocial stress and affected physical health (4–6).

Myopia has become a significant public health problem and is the most common cause of vision impairment in children (7), especially in East and Southeast Asia (8, 9). Insufficient time spent outdoors, and prolonged near-work activities are recognized as important risk factors for myopia (7, 10). Studies have found that the COVID-19 leads to beneficial conditions for behavioral changes in myopia, characterized by reduced outdoor activity and increased near work (11, 12). Children use electronic devices for virtual education, social interaction, and entertainment during the COVID-19 pandemic, with the amount of time Hong Kong children spend on electronic devices increasing from 2.5 h a day to nearly seven hours a day (11, 13). Liu et al. have pointed out that daily use of digital screens was positively related to the prevalence of myopia progression (14). A consequence of the forced lifestyle change during the COVID-19 pandemic would likely have a significant effect on the global burden of myopia (10, 11, 13–15). The purpose was to assess the impact of study-at-home during the COVID-19 pandemic on myopia progression in children by comparing the cycloplegic spherical equivalent, uncorrected visual acuity (UCVA) and axial length (AL). These data will provide important insight into myopia progression.

## Methods

### Study Population

In 2019, three of nearly 20 primary schools in Fuxing District, Handan, Hebei, China were selected for an observational study using a cohort randomization method, which was approved by the ethics committee. Comprehensive and detailed ophthalmic examinations were performed for children of grade 3 in July 2019 and January 2020. In 2020, schools were closed from January to August due to COVID-19. Therefore, the data were collected at the Third Hospital of Handan in August 2020. The children in the exposed group studied at home during the COVID-19 pandemic (January–August 2020) and did not study at home before the COVID-19 outbreak. All participants met the following inclusion criteria: the best-corrected visual acuity of all participants was at least 0.0 Log MAR in both eyes. The exclusion criteria were systemic diseases, former or current eye diseases and injury. Children with atropine or orthokeratology or any condition that might have influenced myopia were excluded.

Stratified cluster sampling was used in the ACES. The ACES is a school-based cohort study mainly designed to longitudinally observe the occurrence and development of myopia in school-aged children. Details of the ACES have been published previously (9). Children in the Anyang Children Eye Study (ACES) matched with children in the exposed group were included in the control group. All children in the control group were not exposed to exposure factors (study at home). A total of 2,850 children in ACES database were the same age and sex as those in the exposed group. According to the matching principles, children in the control were selected from 2,850 children. Matching principles: 1) age, with a difference within ± 0.25 years, 2) sex, 3) UCVA, 4) degree of myopia, and 5) AL, with a difference within ± 0.25 mm. The exclusion criteria were the same as those in the exposed group.

The study was approved by the Ethics Committee and Institutional Review Board of Beijing Tongren Hospital, Capital Medical University. Written informed consent forms were brought home by the children of exposed and control group for their parents' signature, and this study adhered to the principles of the Declaration of Helsinki.

### Procedures

Children in the exposed group underwent a comprehensive and standardized ophthalmic examination to obtain data on UCVA, cycloplegic refraction and AL at baseline (July 2019), at the first follow-up (January 2020) and at the second follow-up (August 2020) in Handan Third Hospital. In addition, a detailed questionnaire was administered to children in the exposed group at the second follow-up.

Children were assessed for distance visual acuity (VA) without spectacles using a logarithmic VA chart (Precision Vision, La Salle, IL, USA) at a distance of 4 m. The chart was retro-illuminated and had 70 tumbling “E” optotypes with five letters on each line. Children were examined monocularly (left eye followed by the right eye); the detailed procedure has been described elsewhere (16). AL was measured by an IOL Master 700 (Carl Zeiss Meditec AG, Jena, Germany). Five repeated measurements were performed, and the average AL value before cycloplegia was obtained. Autorefraction keratometry (KR8800, Topcon, Tokyo, Japan) was used to measure cycloplegic autorefraction. In each child, three drops of 1% cyclopentolate (Cyclogyl, Alcon-Convreur, Rijksweg, Belgium) were administered at intervals of 5 min. If the pupil size was less than 6.0 mm or a light reflex was still present after 30 min, a fourth drop of 1% cyclopentolate was administered, and the examination was repeated after 15 min (17). Three autorefraction readings were taken, and the average was recorded.

The questionnaire used in our study was derived from that in the Anyang Children Eye Study, and the detailed procedure has been described elsewhere (11). To control for interview bias, the questionnaire was administered in a pilot study to determine validity and reliability (7). The questionnaire mainly focused on collecting near-work, outdoor activity-related and online education data, including time (hours/day) spent performing and types of near-work and outdoor activities before and during the study-at-home period. Near-work included homework, reading books, painting, playing chess, using computers, and using mobile phones. Outdoor activities were bicycle riding, running, swimming, playing football, etc. The parents and children were asked about the time (hours/day) spent engaging in and parameters of online education during the study-at-home period. All questionnaires were completed by the children and their parents together. Due to the limited literacy ability of children, the questionnaire was completed by children and parents.

Children who were enrolled in the control group underwent ophthalmic examinations in September 2014, September 2015, and September 2016. In the control group, all students had UCVA (Precision Vision, La Salle, IL, USA) measured at a 4 m distance. The charts were the same for the control and exposed groups. Children were examined monocularly (left eye followed by the right eye); the detailed procedure has been described elsewhere (16). An autorefractor (HRK7000A, Huvitz, Gunpo, South Korea) was used to measure cycloplegic refraction. Each student was first administered one drop of topical anesthetic agent (Alcaine, Alcon) to alleviate discomfort, followed by two drops of 1% cyclopentolate (Alcon) and 1 drop of Mydrin P (Santen, Japan) after a 5-min interval. Thirty min after the last drop, a third drop of cyclopentolate was administered if the pupillary light reflex was still present or the pupil size was less than 6.0 mm. Three readings of spherocylindrical auto-refraction were taken and averaged. An IOL Master (Carl Zeiss Meditec AG, Jena, Germany) was used to measure axial length. Five repeated measurements were taken and averaged before cycloplegia. The detailed procedure has been described elsewhere (9).

### Definitions

The spherical equivalent (SE) was calculated according to the standard formula of the algebraic sum of the dioptric powers of the sphere and half of the cylinder (sphere +0.5 × cylinder). Myopia was defined as an SE ≤ −0.5 D and was classified as low (−3.0 D < SE ≤ −0.5 D), moderate (−6.0 D < SE ≤ −3.0 D) or high (SE ≤ −6.0 D) myopia (18). Emmetrope and hyperopia were defined as an SE between −0.5 D and +0.5 D and greater than +0.5 D, respectively (18).

In the exposed group, the period from July 2019 to August 2020 was divided into two periods: the 7-month period during the COVID-19 pandemic (follow-up period: January – August 2020) and the 7-month period before the COVID-19 outbreak (baseline period: July 2019 – January 2020). Children in the control group did not study at home from September 2014 to September 2016. In the control group, the period from September 2014 to September 2015 was considered the baseline period, and the period from September 2015 to September 2016 was considered the follow-up period.

In the control group, the changes in ophthalmic parameters in one year were calculated as the value in September 2015 minus the value in September 2014 or the value in September 2016 minus the value in September 2015. The changes in ophthalmic parameters at 7 months were calculated by the changes in ocular parameters in a year divided by 12 and multiplied by 7.

### Data Management and Statistical Analysis

Data were entered into EpiData software 3.1 (The EpiData Association, Odense, Denmark), and statistical analysis was performed using an open-source R program (version 4.0.2).

The Shapiro-Wilk test was used to test for the normality of continuous variables. For normally distributed continuous variables, means and standard deviations were used to provide a basic statistical description. Median values and interquartile ranges were used for basic statistical descriptions of non-normally distributed continuous variables. The Wilcoxon rank sum test was used to compare continuous outcomes between the exposed group and the control group; the Wilcoxon signed rank test was used to compare continuous outcomes between period one and period two in the exposed group. Analysis of covariance was used to adjust the influence of axial length change and then make comparison of refraction change between different groups. The difference-in-difference approach was used to analyze the impact of study-at-home. Since large correlation coefficients were observed between the two eyes (*P* < 0.001), only data from right eyes were included in the analyses. Two-sided *p*-values less than 0.05 were considered statistically significant.

## Results

A total of 154 children aged 8.65 ± 0.29 years were included in the analysis. There were 77 children in each of the two groups and 40 (51.95%) males in each group. For the baseline period, 25 children had myopia (25/77) in each of the two groups. There were no significant differences between the two groups in uncorrected visual acuity, spherical equivalent or axial length at baseline (Table 1).

**Table 1:** Baseline ocular characteristics in the exposed and control groups.

	**Control (*N =* 77)**	**Exposed (*N =* 77)**	
**Variables**	**Mean ± SD**	**Mean ± SD**	***P***
Age (Y)	8.65 ± 0.29	8.65 ± 0.29	1.00
Gender, male/female	40/33	40/33	1.00
Uncorrected visual acuity	0.16 ± 0.21	0.16 ± 0.21	1.00
Spherical equivalent (D)	−0.14 ±1.09	−0.26 ± 0.93	0.38
Axial length (mm)	23.21 ± 0.71	23.18 ± 0.72	0.83

*SD, standard deviation*.

**Figure 1:**
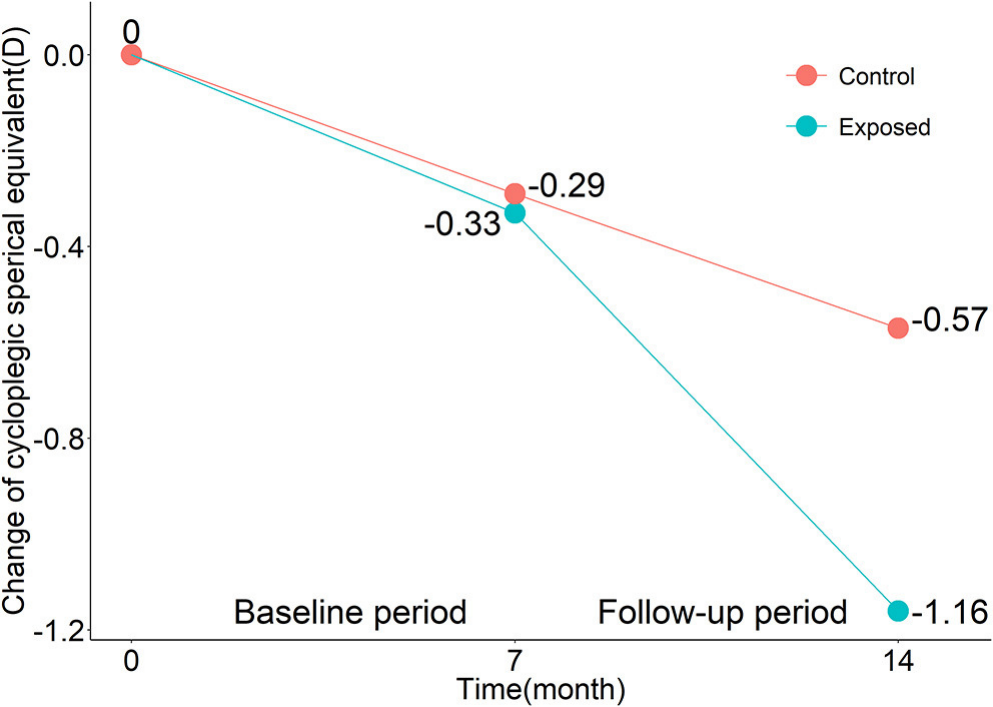
Mean changes in cycloplegic spherical equivalents in the exposed group and the control group over the baseline period and follow-up period.

In the control group at the last follow-up 37 children had myopia (37/77); however, sixty-five children had myopia (65/77) in the exposed group at the last follow-up. No difference was observed between the two groups in the baseline period (*p* = 0.62). In the follow-up period, a significantly larger change in myopia progression in the exposed group (−0.83 ± 0.56 D) than in the control group (−0.28 ± 0.54 D; *p* < 0.001; Figure 1) was observed. The effect of axial length change on myopia progression was not statistically significant by covariance analysis (F = 0.788; *p* = 0.705). In addition, in the exposed group, children had a larger change in myopia progression in the follow-up period (−0.83 ± 0.56 D) than in the baseline period (−0.33 ± 0.46 D; *p* < 0.001; Figure 1). The effect of axial length change on myopia progression was not statistically significant by covariance analysis (F = 0.800; *p* = 0.692). Differences-in-differences estimation of the impact of study-at-home indicated that it accelerated myopia progression (t = −0.567; *p* < 0.001).

There was no significant difference in the myopia progression in the baseline period between the exposed and control groups. However, in the follow-up period, the median myopia progression of the exposed group (−0.75 D) was significantly larger than that of the control group (−0.28 D; *p* < 0.001; Table 2). No differences in the change in UCVA and axial elongation were observed between the two groups (Table 2). In the exposed group, the median (quartile) myopia progression in the follow-up period was −0.75 (−1.13, −0.50) D, which was significantly different than that in the baseline period (−0.38 (−0.63, 0.00) D; *p* < 0.001). The changes in the UCVA between the two periods were not significantly different than those in the exposed group (0.00 vs. 0.01, *p* = 0.059). In the baseline period, the median (quartile) value of axial elongation was 0.20 (0.14, 0.28) mm, which was not significantly different from 0.20 (0.11, 0.38) mm in the follow-up period (*p* = 0.97; Table 2). In the exposed group, no difference in the change of astigmatism was observed (0.06 vs. 0.08, *p* = 0.19).

**Table 2:** Changes in ophthalmic parameters between the exposed and control groups in baseline period and follow-up period.

**Variables**	**Exposed**	**Control**	***P***
	**Median (Quartile)**	**Median (Quartile)**	
Change in spherical equivalent (D)			
Baseline period^*^	−0.38(−0.63, 0.00)	−0.29(−0.58, 0.00)	0.62
follow–up period^†^	−0.75(−1.13, −0.50)	−0.28(−0.50, −0.04)	<0.0001
Change in uncorrected visual acuity			
Baseline period^*^	0.10(0.00, 0.10)	0.06(0.00, 0.17)	0.71
Follow-up period^†^	0.00(0.00, 0.10)	0.05(0.00, 0.13)	0.23
Change in axial length (mm)			
Baseline period^*^	0.20(0.14, 0.28)	0.20(0.12, 0.29)	0.42
Follow-up period^†^	0.20(0.11, 0.38)	0.21(0.12, 0.28)	0.43

* *Baseline period for the exposed group was 7-month period before the COVID-19 outbreak (July 2019 - January 2020) and for the control group was 7 months in 2015*

† *Follow-up period for the exposed group was 7-month period during the study-at-home period (January – August 2020) and for the control group was 7 months in 2016*.

**Figure 2:**
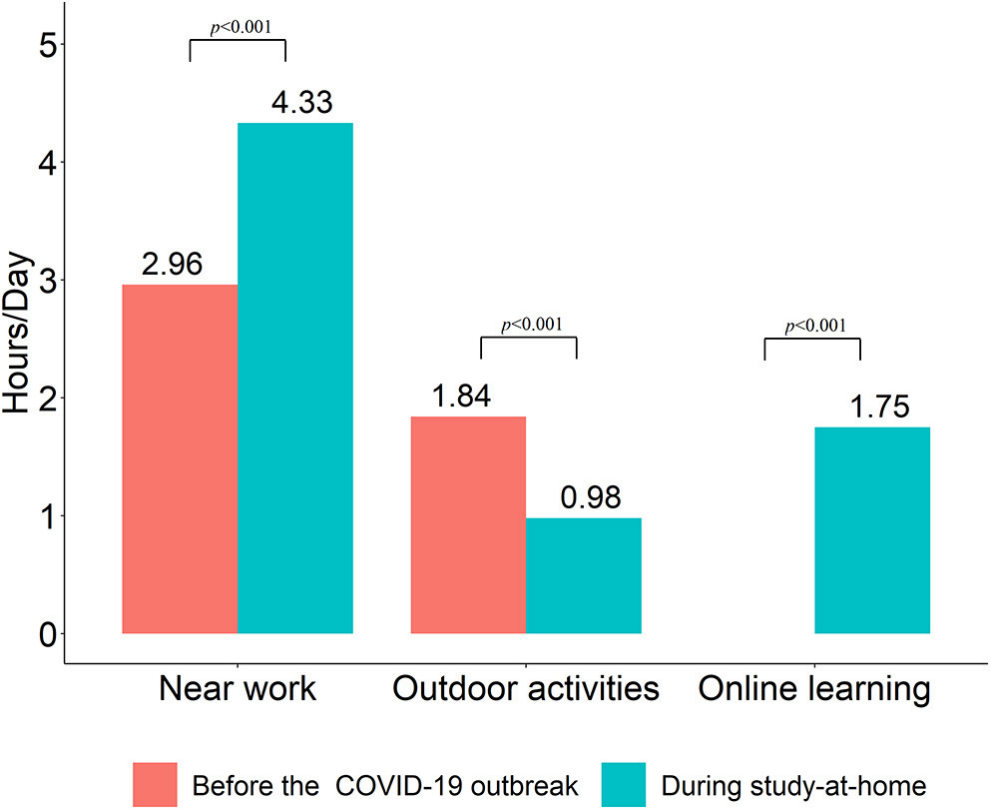
Time spent performing near work and outdoor activities (hours/day) before and during the study-at-home period.

Compared with what was observed before the COVID-19 outbreak, the amount of time spent performing near-work increased during the study-at-home period from 2.96 ± 1.05 hours per day to 4.33 ± 1.04 hours per day (*p* < 0.001). Outdoor activities decreased from 1.84 ± 1.43 hours per day to 0.98 ± 1.01 hours per day (*p* < 0.001). In the control group, schoolchildren from Anyang spent 2.27 hours per day on outdoor activity, which was not significantly different than that in the baseline period of the exposed group (2.27 vs. 1.84; *P* = 0.25). Children in the exposed group spent 1.75 ± 0.71 hours per day engaging in online education during the COVID-19 pandemic. The details are presented in Figure 2.

## Discussion

Concerns have been raised about myopia development in children during study-at-home caused by COVID-19. This study compared data on changes in ophthalmic parameters between the exposed and control groups in the baseline period (pre-COVID) and follow-up period (during COVID study-at-home). Myopia is a serious public health problem (7). In the past few decades, the prevalence of myopia has increased rapidly (7, 8). The study-at-home during the COVID-19 pandemic might have influenced myopia progression in children since it is well known that the change in visual habits may impact the burden of myopia.

A summary of myopia progression in previous studies is shown in Table 3 (13–21). Our study found that myopia progression, at −0.3 ± 0.5 D, at 7 months before the COVID-19 outbreak was similar to those in previous studies in Chinese and foreign children, showing myopia progression of −0.3 D in 6 months or −0.6 D in a year (20, 22–26). Some studies also reported a higher myopia progression rate (21, 27). We believe that this may be related to different genetic and environmental factors as well as differences in subjects' age and the refractive errors in different studies.

**Table 3:** Key results of and myopia progression according to previous studies.

**Reference**	**Location**	**Age, y**	**Sample size**	**Duration, months**	**Average myopia, D**	**Myopia progression, D**
Donovan et al. (20)	China	6–12	85	6	NA	−0.31 ± 0.25 for summer, −0.53 ± 0.29 for winter
Fujiwara et al. (23)	Japan	10–13	92	6	−4.40 ± 1.38	−0.35 ± 0.04 for summer, −0.28 ± 0.06 for winter
Cui et al. (28)	Denmark	8–14	235	6	−2.24 ± 1.39	−0.287 ± 0.266
Gwiazda et al. (24)	USA	6–12	469	6	– 2.54 ± 0.84	−0.35 ± 0.34 for winter, −0.14 ± 0.32 for summer
Yu et al. (21)	China	6–15	900	6	NA	−0.56 ± 0.37
Clark et al. (25)	USA	6–15	NA	12	−2.0 ± 1.5	−0.6 ± 0.4
Wu et al. (27)	Taiwan	6–7	41	12	NA	−0.79 ± 0.38
Larkin et al. (26)	USA	6–15	98	12	−2.8 ± 1.6	−0.6 ± 0.4
Chen et al. (22)	China	8–15	144	12	−3.16 ± 1.13	−0.61 ± 0.31

We found that myopia progression during the study-at-home period was higher than the highest myopia progression during the period before COVID-19 in other studies (21). The changes in visual acuity and axial elongation were not statistically significant; we believe this might be related to the small degree and short follow-up time. The children in this study were in the developmental stage, during which the progression of myopia causes hyperopia peripheral defocus, which causes lengthening of the axis (29).

Previous investigations have reported (4–6) that home confinement during the COVID-19 was associated with the myopia shift. Wang et al. proposed that the age of 6–8 years is the critical period for the development of myopia in children, possibly because younger children's refraction status is more sensitive to environmental changes (15). The relationship between lifestyle changes and myopia has been reported in multiple studies. Liu et al. found that every additional hour of daily digital screen use was associated with an increased risk of myopia progression (5, 14).

Children's lifestyles have changed among children during the COVID-19 pandemic (12, 30). During the study-at-home period, students were away from the school classrooms and engaged in online education, which greatly increased digital screen time. In our study, we found that children spent 1.75 ± 0.71 hours per day on online education and 4.33 ± 1.04 hours per day on near work. In Spanish and Hong Kong, children spent more time on near work, and the students from Hong Kong spent nearly 7 hours/day on digital screens (12, 30). Long-term near work is considered a risk factor for the prevalence and incidence of myopia through increased accommodative demand (8, 10). Digital screen time has been reported to be a risk factor for increased myopia (5, 14, 31). Liu et al. pointed out that extended sedentary engagement with digital devices and psychosocial stress associated with prolonged social isolation were important risk factors for the development of myopia. (6, 14). During the COVID-19 pandemic, the outdoor activities of children and teenagers were reduced because of the various lockdown measures imposed on populations everywhere to contain the spread of the virus. The time of outdoor activities in Handan decreased from 1.84 ± 1.43 hours per day to 0.98 ± 1.01 hours per day. In Hong Kong, the students spent 0.4 hours/day on outdoors (30). A previous meta-analysis has found that outdoor time has a protective effect on myopia onset (32). With the spread of COVID-19, there has been an increase in quarantine dry eye patients all around the worldwide (33). During the COVID-19 pandemic, too much or too little indoor lighting can have an impact on children's vision development (34). In short, prolonged digital device time, dry eye, reduced outdoor activity time, too bright or too dark light and psychosocial pressure may all be risk factors for the progression of myopia.

However, this study has several limitations. The first is selection bias due to a relatively small sample size. Although the sample size was small, the comprehensive and detail ophthalmic examinations were performed. Mydriatic refraction data and collected visual acuity and axial length data were collected and analyzed. Second, the exposed group and the control group were from two cities in different periods, but the two cities were both in Central China. The distance between two cities is approximately sixty kilometers. The diet, climate, socioeconomic status, and culture of the two cities are similar. With all Chinese students studying at home during the COVID-19 outbreak, the best match we found was with Anyang's children's data. The ages of the experimental group and the control group were matched, and the method of difference analysis was used. These minimize the impact of children's growth and development on results. Third, the data on the time and parameters of near-work and outdoor activities were not directly measured but were acquired by questionnaires, which may have led to recall bias. Due to the limited literacy ability of children, the questionnaire was completed by children and parents. To minimize recall bias, the questionnaire should be completed by children, and the parents should confirm the children's answers. Fourth, the auto-refractors of control group and exposure group were different, which may affect the measurement results. In the future, we will study and compare the measurement errors of the two devices.

## Conclusions

The natural experiment provided the empirical evidence that children who studied at home are at high risk of myopia progression. Home quarantine and study-at-home during COVID-19 may worsen the global burden of myopia. Considering the persistence of COVID-19 and potential risk of study-at-home order again, continuous attention should be given to the occurrence and development of myopia.

## Data Availability Statement

The original contributions presented in the study are included in the article/supplementary material, further inquiries can be directed to the corresponding authors.

## Ethics Statement

The studies involving human participants were reviewed and approved by the Medical Ethics Committee of the Beijing Tongren Hospital. The patients/participants provided their written informed consent to participate in this study. Written informed consent to participate in this study was provided by the participants' legal guardian/next of kin.

## Author Contributions

DM was responsible for collecting and analyzing data, interpreting results, and writing the abstract and the paper. SW, S-ML, XY, KC, JH, XP, RY, and JF contributed to reviewing of the data, interpreting results, and writing the tables and figures. AG, Z-BJ, and NW contributed to the design of the study, review, and feedback on the paper. All authors contributed to the article and approved the submitted version.

## Funding

This work was supported by the Fund Sponsorship of the Capital Public Health Project (Z171100000417017), the Beijing Natural Science Foundation (JQ20029), the Capital health research and development of special (2020-2-1081), the National Natural Science Foundation of China (82071000).

## Conflict of Interest

The authors declare that the research was conducted in the absence of any commercial or financial relationships that could be construed as a potential conflict of interest.

## Publisher's Note

All claims expressed in this article are solely those of the authors and do not necessarily represent those of their affiliated organizations, or those of the publisher, the editors and the reviewers. Any product that may be evaluated in this article, or claim that may be made by its manufacturer, is not guaranteed or endorsed by the publisher.
